# The Midlands Liver Research Alliance - A partnership to optimise obesity-related liver disease research: targeting areas of high incidence and underserved communities

**DOI:** 10.3310/nihropenres.13784.1

**Published:** 2024-12-12

**Authors:** James King, Guruprasad Aithal, Louisa Herring, Scott Willis, Dimitris Papamargaritis, Kerry Hulley, Melanie Davies

**Affiliations:** 1 Nottingham Digestive Diseases Center, School of Medicine, University of Nottingham, Nottingham, NG7 2UH, UK; 2 NIHR Nottingham Biomedical Research Centre, Nottingham University Hospital NHS Trust, and the University of Nottingham, Nottingham, UK; 3 NIHR Leicester Biomedical Research Centre, University Hospitals of Leicester NHS Trust, and the University of Leicester, Leicester, UK; 4 National Centre for Sport and Exercise Medicine, School of Sport Exercise and Health Sciences, Loughborough University, Loughborough, England, LE11 3TU, UK; 5 Diabetes Research Center, University of Leicester, Leicester, UK; 6 University Hospitals of Leicester NHS Trust, Leicester, England, UK

**Keywords:** Liver, metabolic dysfunction-associated steatotic liver disease (MASLD), physical activity, exercise, nutrition, education, patient-reported outcomes

## Abstract

**Background:**

The prevalence of liver disease is rising in the United Kingdom (UK), with obesity underpinning surging metabolic dysfunction-associated steatotic liver disease (MASLD). MASLD is associated with an increased cardiometabolic risk, particularly when co-existing with type 2 diabetes. Progression to metabolic dysfunction-associated steatohepatitis (MASH) with hepatic fibrosis represents a clinical milestone strongly linked to serious liver disease and mortality.

Therefore, clinically meaningful and sustained weight loss (≥10%) is a primary therapeutic target for patients with MASLD. Unfortunately, this is difficult for most people who adopt traditional lifestyle approaches. However, new obesity pharmacotherapies hold promise in MASLD, given their ability to produce dramatic weight loss (10–25%) and improve cardiometabolic health. Questions remain about the ability of these agents to improve liver fibrosis and patient-reported outcomes/quality of life in patients with advanced liver disease.

**Methods:**

Led from the Midlands (UK) but with national representation, we developed a network of stakeholders (clinicians, academics, third-sector, industry, and PPIE representatives) with an interest in obesity-related liver diseases. This network was called the Midlands Liver Research Alliance (MLRA), which sought to 1) establish a PPIE stakeholder network, 2) identify research priorities, and 3) map the network infrastructure and expertise. Health inequalities within liver disease are a core priority within the MLRA.

**Results:**

The MLRA developed a large PPIE stakeholder network in collaboration with other local liver partnerships. These networks facilitated the identification of key research priorities that led to three NIHR funding applications. Priorities centered around: 1) the importance of patient-centered outcomes in obesity-related liver disease research; 2) the potential of glucagon-like peptide 1 (GLP-1)-based obesity pharmacotherapy in alcohol use disorder; and 3) early identification and management of liver disease in primary care/community.

**Conclusions:**

The MLRA has created a multidisciplinary hub of research expertise in obesity-related liver disease. This foundation provides a springboard for research activities in this area.

## Plain Language Summary

Liver disease is a major cause of death worldwide and is becoming more common each year. This is mostly due to fat in the wrong place in the liver, which can cause liver cirrhosis (scarring). The causes include obesity, type 2 diabetes, and excessive alcohol consumption. These problems are often linked to lifestyle choices such as not exercising enough and eating unhealthy foods.

The Midlands Liver Research Alliance (MLRA) is a partnership made up of researchers, health care professionals, people with liver disease, and charities, funded by the NIHR to identify gaps in liver disease care and research in areas most in need. Leicester, Nottingham, and Birmingham’s Biomedical Research Centers are leading experts in liver and obesity research and have led the MLRA partnership.

The MLRA established an inclusivity and engagement network, identified research experts and centers, and set important liver research priorities. The three liver research priority areas the MLRA has and will continue to focus on are:

*Experimental medicine -* research focusing on the impact of obesity medications and lifestyle activities such as exercise on this type of liver disease.

*Addressing health inequalities in liver disease -* within our diverse region, working with expert centers and charities to reduce inequalities relating to ethnicity, socioeconomic position, and coastal communities within liver disease.

*Liver disease management in primary care*: work with GP practices to provide liver disease education for healthcare professionals to help early identification and management. Provide education and support for people at risk of liver disease.

The MLRA uses this extensive knowledge gathered from MLRA priority setting activities and collaborative activities with other liver partnerships to inform future research and continue collaborations in this important area.

## Background

In Western nations, liver disease is the only chronic condition demonstrating a yearly increase in prevalence, making it the 5 ^th^ leading cause of death after heart disease, cancer, stroke, and respiratory disease. A major contributor to this trend is the surge in metabolic dysfunction-associated steatotic liver disease (MASLD) and metabolic dysfunction-associated steatohepatitis (MASH). The prevalence of these conditions in Europe is high, estimated at 32% and 4% (general population), respectively ^1^. High levels of obesity and type 2 diabetes mellitus (T2DM) underpin these conditions, which increases the risk of cardiovascular disease (CVD) and reduces life expectancy ^2^.

MASLD is a generic term encompassing a spectrum of pathological hepatic phenotypes. In obesity, excess accumulation of liver fat is the primary insult associated with dysglycemia and heightened cardiovascular risk. Over time, the development of hepatic inflammation and hepatocyte injury indicates a more advanced and aggressive phenotype with a more deleterious prognosis ^2^. Specifically, the subsequent development of hepatic fibrosis, sometimes termed fibrotic MASH, represents a clinical milestone in which progression to CVD and end-stage liver disease is exaggerated, multiple long-term conditions (MLTC) are common, and health-related quality of life is poor.

Therefore, weight loss and long-term weight loss maintenance are the primary targets in the management of MASLD ^3^. However, lifestyle approaches (diet and physical activity) are typically unable to facilitate clinically meaningful and sustained weight loss in most people. The field of anti-obesity pharmacotherapy has ignited in recent years, led by glucagon-like peptide-1 (GLP-1)-based therapies ^4^. Following the STEP phase 3 trial program, the GLP-1 receptor agonist (GLP-1 RA) semaglutide (2.4 mg) was licenced in the UK for the treatment of obesity. These trials showed that semaglutide induces clinically relevant weight loss in people with obesity ^5^, which is sustained while the treatment is maintained ^6^. More recently, the GLP-1 / gastric inhibitory polypeptide (GIP) receptor co-agonist tirzepatide (at doses of 5, 10, and 15 mg) was approved for obesity therapy in the UK. The weight loss induced by tirzepatide 15 mg is greater than semaglutide 2.4 mg ^7^. Moreover, in individuals with liver disease, tirzepatide was recently shown to resolve MASH in two-thirds of individuals and reduce fibrosis by more than one stage in approximately half ^8^. With tirzepatide, questions remain about whether longer treatment duration (to permit maximum weight loss) is able to provide greater hepatic benefits and the impact of therapy on key patient-reported outcomes, including functional health. Moreover, the extent to which the benefits of pharmacotherapy can be potentiated by lifestyle (exercise and diet) remains unclear.

In addition to obesity, GLP-1-based therapies may have wider applications in liver disease, specifically in the treatment of alcohol addiction (alcohol use disorder [AUD]) ^9^. In the UK, alcohol-related liver disease (ARLD) has increased sharply in recent years and is difficult to treat. Moreover, half of the people with AUD also have obesity ^10^, which potentiates adverse health effects, including mortality ^11^. The pernicious interaction between alcohol consumption and obesity-related liver disease was formally recognized in the recent update to the liver disease nomenclature, with the term ‘metabolic dysfunction and alcohol-associated liver disease’ (MetALD) ^12^. In a global Delphi consensus study, this term was chosen to indicate patients with MASLD who regularly consumed alcohol (140–350 g/week women, 210–420 g/week men) without a diagnosis of ARLD. While GLP-1 therapies reduce appetite and food intake through central effects in the hypothalamus, wider impacts on central reward-related pathways may reduce craving for alcohol ^9^. At present, a range of pre-clinical and observational studies have shown the potential of GLP-1-based therapies in AUD. However, further experimental research is needed to demonstrate its efficacy in humans.

The health and economic burden of liver disease is heavily biased towards advanced stages ^13^. Modelling of future disease prevalence indicates that specialist hepatology and gastroenterology services will experience increasing strain ^14^. Therefore, risk factor identification and early intervention are becoming increasingly important for patients and health services. Primary care is central to this ambition, yet liver disease is often not a priority for GPs and AHPs in general practice. Besides competing priorities, this may be due to a lack of knowledge, confidence, and ongoing training on liver disease in primary care ^15, 16^. This may also be related to the absence of bespoke therapies in at-risk patients. A better understanding and awareness of liver disease, for healthcare professionals and patients with risk factors, is needed in a switch to a more prevention-focused mindset. 

The prevalence and health burdens associated with obesity-related liver disease disproportionately affect people living in poorer regions, including minority ethnic groups and coastal communities. Notably, the Chief Medical Officers’ annual report in 2021 highlighted high rates of preventable diseases and low life expectancy in coastal regions, encouraging strategies to address health inequalities ^17^. Moreover, individuals from South Asian communities are known to more readily develop obesity-related conditions such as T2DM and MASLD ^18^, underscoring the importance of research in areas of greatest need.

## Project aims & objectives

The overarching aim of our NIHR liver partnership award was to explore issues and identify research questions surrounding obesity-related liver diseases and associated health inequalities in the UK. To do this, we set out to establish a cross-sector, multidisciplinary research partnership based in the Midlands, but with support from collaborators nationally and internationally. Our specific objectives were as follows:

1. Cultivate a sustainable public and stakeholder engagement network that focuses on underserved populations.
2. Identify and define obesity-related liver disease research priorities.
3. Establish partners to scope and facilitate the development of a core set of patient-reported outcomes.
4. Generate an effective research implementation framework for clinical practice.
5. Create a network of obesity-related liver disease satellite research sites to provide increased research capacity, capability, and knowledge mobilization in areas of need.
6. Offer training to develop obesity-related liver disease clinical and allied health professional (AHP) research specialists and provide training around cultural competencies and underserved populations.
7. Develop cross-infrastructure mentoring and mobilization of research best practices.

## Patient and Public Involvement / Engagement

Our research team first engaged with our PPIE networks during the development of our NIHR liver partnership funding application. Initially, the core research team liaised with individuals who had previously acted as PPIE representatives for research. Through a series of meetings, these individuals contributed to our initial application by helping to steer the prospective partnership focus.

As explained in detail below, a wider pool of PPIE members and stakeholders become a core part of our partnership. Perhaps their most important contribution to the partnership was their input into the focus and development of funding applications conceived by the partnership. This included the provision of opinion and experience that helped to shape the key research questions, study design and recruitment approaches. For each application submitted, at least one PPIE member agreed to become part of the study team and serve on the trial steering committee.

## Activities (1) – PPIE & stakeholder inclusivity & engagement

After receiving the award, an Operational and Engagement lead was appointed to oversee the day-to-day activities associated with the MLRA to create a successful partnership. A series of regular (bi-monthly) core working group meetings were organized and held with partnership investigators, wider stakeholders, and two individuals with a lived experience of obesity-related liver disease (PPIE representatives).

Initially, these meetings strongly focused on the evolution and development of the partnership. The website https://midlands-liver-research.squarespace.com and branding in the form of a logo were designed with the support of PPIE members. The website housed information for patients, members of the public, and healthcare professionals relating to MASLD (e.g., risk factors, risk reduction, stigma, treatments, nomenclature, and interviews with Prof. Guru Aithal and patients with lived experience). It also contained an interactive map of where the partnerships’ national collaborators were located and their specialities.

Core members of the MLRA team attended the launch of the four Nottingham Biomedical Research Center (BRC) partnerships in which the MLRA formed a part. This event was attended by diverse groups, including those associated with local partnerships (academics and clinicians), community and organizational stakeholders, and patients with a lived experience of liver disease. At this meeting, Dr. James King presented the rationale behind the MLRA partnership, and with Prof. Guru Aithal, discussed co-elaborative opportunities with leads from the Nottingham partnerships. Round table discussions concluded the day with key discussions, including improving inclusivity in liver research, ideas of how to build national and local liver PPIE groups, and what outcomes are important in both alcohol- and obesity-related liver research.

The MLRA launch meeting and ‘Discovery’ priority setting events included people with lived experience, public members, clinicians, and third-party sector organizations covering the whole of the East Midlands. At this collaborative Discovery Event, each of the four Nottingham BRC liver partnerships presented their work to ascertain interest and seek representatives to join one or more of the partnerships, to become empowered, and to enable the development of future liver research proposals. Training on PPIE was also offered in this research. The partnership collaboration event gave everyone the opportunity to talk with other delegates and liver specialists in attendance. The MLRA recruited new PPIE members as a result of this meeting.

Core MLRA members were invited to attend other partnership launch meetings, such as the ALPINE partnership, where all the partnerships were outlined, providing an opportunity for networking and, where possible, collaborative work. During the 18-month term of the partnership, the MLRA core members attended other meetings, such as the British Association of Liver Disease (BASL), British Society of Gastroenterology (BSG), and the MASLD Special Interest group (SIG). These meetings provided an opportunity to update on our progress, with whom we were collaborating, any issues we were experiencing within the partnership, and shared best practices. These meetings were attended by other partnerships, national therapy area specialists, NIHR members, and charity organizations, such as the British Liver Trust. Other topics frequently discussed were the lack of a routine primary care pathway, associated stigma with obesity-related liver disease, and potential issues with the new definitions and nomenclature for people living with MASLD and how this could be integrated within future public awareness campaigns. It also provided the opportunity to discuss potential new future research proposals and whether there would be overlaps or collaborations with other partnerships or working groups.

In conjunction with the Leicester BRC communication manager, a media and marketing plan was developed for the MLRA. Press releases were arranged in a timely manner, highlighting who we are, how you can get involved with the MLRA, and promoting understanding of MASLD and associated risk factors. The audiences that these were released were Leicester, Nottingham, and Birmingham themes and platforms, central members of the core PPIE training operations within Leicester and Nottingham, University Hospitals of Leicester colleagues, GP/primary care, and the public. Part of the campaign included broadcasts through the popular South Asian radio network Sabras radio, increasing awareness, and the risks associated with MASLD during the local GP’s health-related segment.

At the core of our partnership was a focus on PPIE. We established PPIE networks within Leicester and Lincolnshire, including people with lived experience. Some of the patient and public groups consisted of Lincolnshire **H**ealthier **A**ging **P**atient and **P**ublic **I**nvolvement Group (HAPPI), Leicester's Dance and Health group, sports clubs including Leicester City football in the community, Sharma Women Centre, Lincolnshire National institute for Rural Healthcare. These groups determined what activities were important to these communities and how MLRA would support them with increasing knowledge around MASLD to share within their communities. As a result, a ‘Language Matters’ event was held to disseminate the new agreed terminology and highlighted the stigma and perceptions around MASLD, which is now being used to inform language used in further research proposals. The MLRA also provided further researcher PPIE training and developed PPIE representatives who have gone on to co-applicant roles for the subsequent research proposals.

The Nottingham community liver pathway became another important pillar of our PPIE network. Led by Prof. Guru Aithal and Prof. Neil Guha, clinicians in Nottingham set-up this pathway to proactively identify individuals with progressive liver disease in primary care. Patients with risk factors for liver disease (e.g. obesity, T2DM, elevated liver enzymes) are proactively invited for a Fibroscan™ test at a local GP practice or hospital. Those found to have elevated liver stiffness are subsequently invited for a consultation with a hepatologist for further investigation, while others are provided with advice about risk factor management. Members of the MLRA met with clinicians leading this pathway to discuss research priorities. They subsequently attended a series of Fibroscan™ clinics to talk to patients with risk factors and nurses conducting the scans. Investigators also visited hepatology clinics to speak with patients with more progressive disease and the hepatologists leading the clinics. A wealth of knowledge and insight was gained from these sessions. A key element of the success was the ‘live PPIE’ element, where with patients’ consent, investigators were able to sit-in clinics and then talk with patients immediately after their appointment. This was often in the presence of the nurses and doctors, effectively creating mini focus groups – leading to rich discussion. Information gathered from these sessions directly led to two of the funding applications submitted from the MLRA.

## Activities (2) – Research strategy & priority setting

With support from our established PPIE networks and wider partners (academic and non-academic stakeholders), research priorities were identified that would form the basis of funding applications for Stage 2 in the NIHR Liver Call. Published lists of research priorities supported this process. A proforma was circulated among our national collaborators to encourage individuals to put forward research ideas that could be developed during the course of the partnership with support from across the network. Ideas were subsequently shared with the network, with the opportunity for interested parties to express their interest in applications. Core working groups were subsequently established around these proposals, which were then developed and refined over the course of the partnership. To date, this process has led to three stage 1 funding applications submitted to the NIHR Liver Call, as detailed below. 

### 1. NIHR Efficacy, Mechanism & Evaluation (EME)

***Optimising physical function & liver health for patients with fibrotic MASH: efficacy and mechanism of tirzepatide & functional training***

Joint Co-Is: Dr. Dimitris Papamargaritis (University of Leicester), Prof. Guruprasad Aithal (University of Nottingham).

Co-investigators: Dr. James King (Loughborogh University), Prof. Melanie Davies (University of Leicester), Prof. Neil Guha (University of Nottingham), Dr. Lynsey Coreless (Hull University Teaching Hospitals NHS Trust), Dr. Aravamuthan Sreedharan (United Lincolnshire Hospitals NHS Trust), Prof Tom Yates (University of Leicester), and Dr. Louisa Herring (University Hospitals of Leicester NHS Trust).

Summary

Information gathered from PPIE activities undertaken within the Nottingham community liver pathway directly informed this application. The key message taken from this interaction was that whilst patients are attending for investigation into their liver health, the most important priorities for patients themselves was their weight and day-to-day mobility. In essence, patients wanted more support with weight loss, as standard lifestyle advice was deemed ineffective. Patients also wanted support to get around more easily and to maintain independence. This application was therefore developed to simultaneously tackle these two patient priorities. A core theme embedded in this application was a focus on Patient Reported Outcomes which have traditionally been neglected in liver disease research ^19^.

A core working group was established involving academics with expertise in obesity medicine, diabetes, hepatology, physical activity, physical function, behavior change, and clinical trials. PPIE support was provided by the NIHR Leicester BRC team and an expert by experience. The protocol for this application was refined through several online and in-person meetings. Application development was led by Dr. Dimitris Papamargaritis and Dr. James King, with ongoing mentorship from Prof. Guru Aithal and Prof. Melanie Davies. 

The study proposed within this application seeks to determine the impact of obesity pharmacotherapy (tirzepatide), with and without concomitant exercise training, on objective measures of physical function and liver health in patients with MASH and advanced fibrosis. Tirzepatide is a pharmacotherapy initially licenced for T2DM in the UK and has been approved by NICE guidelines for this indication. More recently, tirzepatide has been approved for the treatment of obesity, with NICE guidelines surrounding its use for this indication currently in development (at the time of writing after this statement). Tirzepatide is an attractive therapy in the context of obesity-related liver disease due to its capacity to induce dramatic weight loss, reduce liver inflammation, and potentially reduce liver fibrosis ^8^. Exercise training may optimise body composition during weight loss and improve strength, endurance and mobility ^20^. Together, we hypothesise that the combination of tirzepatide and exercise training may provide the greatest benefits for patients with obesity and mild-to-moderate liver fibrosis.

Mechanistic sub-studies were proposed within this application to specifically investigate changes in organ fibroinflammation and skeletal muscle quality. These mechanistic elements were developed in partnership with experts in bioimaging at the University of Nottingham (Sir Peter Mansfield MRI Center) and the University of Leicester (skeletal muscle biology group).

As part of our partnership’s capacity-enhancing agenda, particularly in areas of low research activity and clinical need (from a liver perspective), we solicited the expertise of the Lincoln Clinical Trials Unit (CTU) to support this bid. The Lincoln CTU is a new unit that receives support from external CTUs. Our trial has provided an opportunity for Lincoln to enhance their skills, knowledge, and experience of large, multicentre CTIMPs.

### 2. NIHR Efficacy, Mechanism & Evaluation (EME)

***A randomised placebo-controlled superiority trial of GLP-1 receptor agonist for the treatment of alcohol use disorder in people with chronic liver disease and obesity with embedded mechanistic study: Controlling Unhealthy Relationships with Both alcohol and obesity (CURB trial)***

Joint Co-Is: Dr. Ashwin Dhanda (University of Plymouth), Prof. Guruprasad Aithal (University of Nottingham).

Co-Investigators: Dr. Mohsan Subhani (University of Nottingham), Dr. James King (Loughborough University), Prof. Siobhan Creanor (University of Exeter), Dr. Fiona Warren (University of Exeter), Prof. Penny Gowland (University of Nottingham), Prof. Melanie Davies (University of Leicester), Dr. Stephen Kaar (Greater Manchester Mental Health NHS Foundation Trust), Mr. Andrew Wragg (Nottingham University Hospitals NHS Trust), Dr. Louise Paterson (Imperial College London), Dr. Tony Goldstone (Imperial College London), Ms. Melinda King (PPIE Representative), and Mr. Justin Greenwood (PPIE Representative).

Summary

Within our partnership, members of our team actively engaged with other NIHR liver partnerships in the process of developing research priorities and funding proposals. These included partnerships focusing on alcohol-related liver disease (ARLD), hosted in Nottingham (NIHR155469) and Plymouth (NIHR154191). ARLD is the commonest form of liver disease in the UK. Mortality from ARLD is rising with half of affected individuals suffering with alcohol use disorder (AUD). This is difficult to treat with current therapies. Recent evidence shows that half of people with AUD also have obesity ^10^. This is problematic as obesity drastically worsens the prognosis for people living with both AUD and obesity ^11^. Tackling obesity within AUD was the 5 ^th^ leading priority identified within the Nottingham ARLD partnership ^21^. Moreover, treatment of AUD in people with ARLD was the number 1 research priority in the 2016 James Lind Alliance Priority Setting Partnership. A core working group, spanning 3 liver partnerships (NIHR154191, NIHR155530, NIHR155469), was therefore set-up to develop a proposal for an intervention to combat AUD and obesity simultaneously. This group involved people with expertise in hepatology, obesity, addiction, clinical trials and psychoneurology. PPIE support was provided by the NIHR Nottingham BRC team and two experts by experience.

This application centred on the use of GLP-1 RA as a simultaneous treatment for obesity and AUD. Specifically, semaglutide is a GLP-1RA which is licenced for use in T2DM and obesity in the UK. At present, pre-clinical studies show that GLP-1 therapy reduces alcohol consumption in animal models ^22^. An expanding body of observational data has also identified associations between GLP-1RA use and reduced alcohol consumption ^22^. Experimental trials show that GLP-1 based therapies may have beneficial impacts in a range of addiction disorders ^9^. A sub-analysis in one trial showed that exenatide reduced alcohol consumption in people with AUD and obesity ^23^.

In patients with obesity and AUD, our application therefore proposes a nine-month, multi-site RCT in the UK, comparing the effect of semaglutide and placebo on drinking behaviour and body weight. An embedded mechanistic sub-study will investigate central mechanisms of action of semaglutide on alcohol consumption.

### 3. NIHR Health Service Deliver Research (HSDR)

***A primary care-level intervention to support metabolic dysfunction-associated steatotic liver disease (MASLD) and related co-morbidities***

Joint Co-Is: Dr. Louisa Herring (University Hospitals of Leicester NHS Trust), Dr. Michelle Hadjiconstantinou (University Hospitals of Leicester NHS Trust).

Co-Investigators: Prof. Melanie Davies (University of Leicester), Dr. James King (Loughborough University), Prof. Guruprasad Aithal (University of Nottingham), Prof. Lynsey Coreless (Hull University Teaching Hospitals NHS Trust), Prof. Neil Guha (University of Nottingham), Prof. Joanna Morling (University of Nottingham), Prof. Laura Gray (University of Leicester), Prof. Sam Seidu (University of Leicester), Mrs. Julia Burdon (University Hospitals of Leicester NHS Trust), Dr. Clare Gillies (University of Leicester), Mrs. Tina Mitchell (PPIE Representative).

Summary

The need to optimize clinical pathways for patients with liver disease was identified as a key priority in the early phases of our partnership. This issue emerged through interactions with PPIE representatives across our networks, as well as from conversations with clinicians. Greater clarity was provided through events hosted by the ALPINE partnership (NIHR155210), which contrasted current versus idealized pathways in liver disease. One of the most important problems is that the early signs of liver disease are often missed in primary care, particularly obesity-related liver disease (MASLD and MetALD). This leads to patients presenting to hospital with acute problems that are more severe and difficult to treat. Moreover, the majority of the economic burden associated with liver disease is heavily biased towards the treatment of advanced disease pathology ^13^. In addition, at present, few resources exist to support patients in self-management of their condition. In fact, our PPIE work showed that most people have very poor knowledge of liver disease, and those at risk often desire more information. Recent changes in the nomenclature of liver disease may have magnified this issue.

Academic literature suggests that liver disease is not well understood in primary care; therefore, patients with risk factors are rarely identified ^15^. This is despite evidence from local community liver pathways demonstrating the high burden of liver disease in the community, particularly among people with a combination of obesity and T2DM ^24^. This burden is likely to be exaggerated in poorer communities, including coastal areas, and in those with a high prevalence of ethnic minority groups. As a result of the MLRA, a working group was set up to develop a project to tackle these problems. The group was composed of experts in hepatology, obesity, T2DM, primary care medicine, public health, behavioral change, and structured education. This group decided to focus on two issues: 1) education of GPs and AHPs in primary care, and 2) self-management of patients with risk factors for MASLD in primary care.

To tackle our first priority, our partnership linked-up with the EDEN team hosted within the Leicester Diabetes Center. EDEN is internationally recognized for its structured education programs in diabetes and MLTCs. Together, we developed a prototype educational intervention for GPs and AHPs in primary care, focusing on chronic liver disease. The intervention, called ‘Simplifying Liver Disease,’ was subsequently piloted across Leicestershire and Lincoln (n = 34). Knowledge and confidence questionnaires were used to obtain feedback to facilitate refinement of the intervention.

To address our second priority, we worked with colleagues leading the DESMOND programs in Leicester, which is a suite of patient education and self-management tools for patients with diabetes. The DESMOND team helped our group design a prototype for a self-management resource focused on supporting patients identified as being at risk for obesity-related liver disease. 

As part of our partnership, steps were taken to explore the need for a core outcome measurement in effectiveness trial (COMET) in obesity-related metabolic liver disease. This initiative’s aim was to facilitate the development and application of ‘core outcome sets’, which are an agreed standard set of outcomes that should be measured and reported in all clinical trials in specific areas of health, or healthcare. A COMET working group workshop comprising experts in liver, COMETs, patient-reported outcomes, and experts from the Centre for Patient Reported Outcomes Research (University of Birmingham) discussed the method of developing the core outcome sets, its scope, any relevant stakeholders, the process and discussed any already available core outcome sets in this area. After further discussions with experts and exploration into COMETs already published in this area ^25^, it was decided that a new COMET would not add much more than already exists, particularly around patient-reported outcome measures, such as quality of life. Through extensive PPIE work we identified the gap in health care professional training. It was therefore decided that the development of HCP training and a small pilot would be of more benefit and better value for money output from our partnership. This HCP training development and pilot has underpinned the HSDR funding application submitted.

## Activities (3) – Partnership capacity and capability mapping

**Figure 1.**
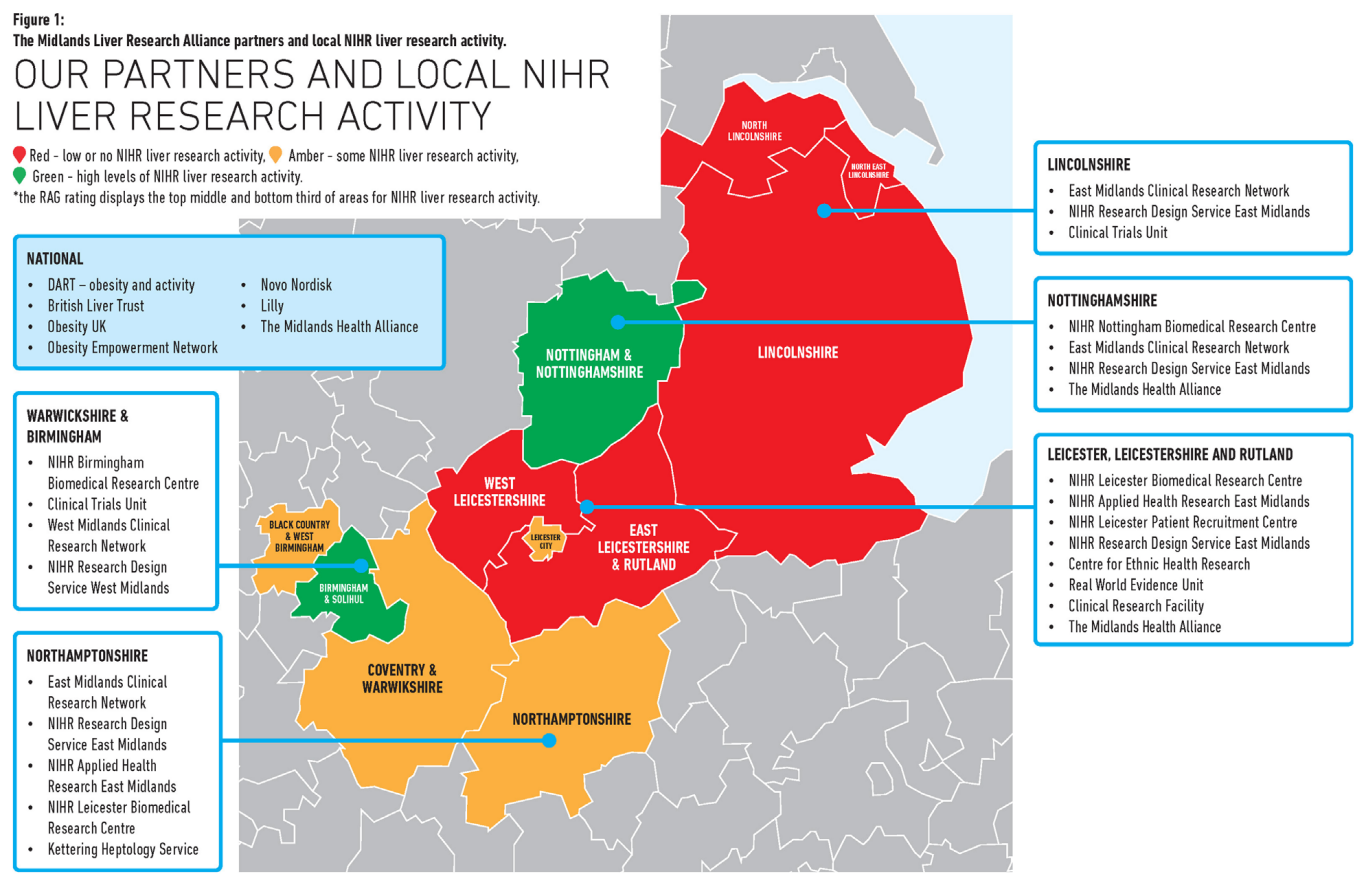
The Midlands Liver Research Alliance partners and local NIHR liver research activity.

A key aim of our partnership was to develop a multidisciplinary network of stakeholders, concentrated in the Midlands yet reaching out nationally, focused on obesity-related liver disease. In all, our network included 89 individuals involving clinicians, academics, PPIE representatives, and individuals working for relevant third-sector organisations. Expertise span obesity, lifestyle (diet and exercise), hepatology/gastroenterology, clinical trials, basic science, and research implementation. At an organisational level, our network now spans three NIHR BRCs (Leicester, Nottingham, Birmingham), three CTUs (Leicester, Nottingham, Lincoln), two clinical research networks (East & West Midlands) and the Leicester, Leicestershire, and Rutland Clinical Research Facility (see Figure 1).

This network of individuals helped define the research needs outlined in the previous section. Within this, the need to train general practice staff (GPs and AHPs) in liver disease prevention and management also emerged as a core clinical priority. Collaborative work with local ICBs (Lincolnshire and Leicester, Leicestershire, and Rutland) enabled the development and pilot testing of our ‘Simplifying Liver Disease’ education intervention. This intervention was pilot tested in 16 GP practices across Lincolnshire and Leicestershire, involving 34 healthcare professionals.

In relation to infrastructure and capacity to undertake multi-site research, mentorship became a key priority. In particular lead investigators within our funding applications received close mentorship from the MLRA co-directors (Prof. Melanie Davies and Prof. Guruprasad Aithal). To this end, each of our NIHR EME applications had co-leads, with Prof. Davies and Prof. Aithal supporting less experienced colleagues. 

Our desire to enhance local research capacity was central to our decision to work with the Lincoln CTU for one of our NIHR EME applications. The Lincoln CTU is a new CTU currently pursuing full accreditation. The MRC CTU provides external support for its expanding portfolio of research. To date, however, the Lincoln CTU has less experience with multi-site CTIMPs. Our application has provided an opportunity to help the Lincoln CTU upskill in this area, with additional support available from our local CTUs (Leicester and Nottingham).

Our funding applications also focused on scoping of research infrastructure across our network. Equipment and expertise in relation to MRI and exercise training interventions are pertinent examples. Mechanistic sub-studies within our EME proposals include plans to harmonise MRI procedures across research sites (Nottingham, Leicester, Liverpool). We have identified the work needed to harmonise and validate processes across MRI scanners. The funding to undertake this work has been included in our application. Within our scoping, we found that many research sites in our network do not have the equipment, facilities, or trained staff to undertake exercise training interventions. To support our funding applications, we have therefore designed new protocols allowing interventions to be completed without specialised resources or the need for high levels of staff training.

## What's next / future plans

With the cessation of funding for the liver partnerships, the MLRA will be incorporated as an entity within the NIHR Leicester (lifestyle theme) and Nottingham BRCs (gastrointestinal and liver themes). With oversight provided by Prof. Davies and Prof. Aithal, our focus on obesity-related liver disease will be steered forward by partnership co-investigators.

In the immediacy, our focus will depend on the outcome of our stage 1 funding applications to the NIHR Liver Call. Additionally, we envisage our research and practice efforts centring on three key areas in the short-to medium-term:

### 1. Experimental medicine

Through our local NIHR BRCs, we intend to maintain a core focus on human experimental research – focused on treatment efficacy and underpinning mechanisms. This work is likely to center on drug-lifestyle (exercise and diet) interaction within the context of MASLD. The treatment of MASLD with drug combinations e.g. obesity pharmacotherapy and anti-fibrotic therapies may also be investigated. Members within our network have an accelerator award to develop a platform trial in obesity. If successful, this infrastructure will facilitate the aforementioned experimental research. This work will also be facilitated by know-how gleaned from ongoing phase 2 and 3 trials (locally conducted) relating to GLP-1 / glucagon RA and triple agonists (GLP-1 / GIP / glucagon RA) therapies.

### 2. Addressing health inequalities in liver disease

We will continue to work with the Centre for Ethnic Health Research and the British Liver Trust to combat health inequalities relating to liver disease. Within our region, inequalities relating to ethnicity and coastal communities are particularly prominent. Specially, in the Midlands we have some of the most ethnically diverse communities in the UK, particularly within the East (Leicester) and West (Birmingham) Midlands. Moreover, Lincolnshire has many deprived areas with high levels of obesity and T2DM (MASLD risk factors) across the wider region. 

The aforementioned areas are underserved with regards to health care, whilst cultural / religious / socioeconomic factors may create additional barriers to engagement in relation to liver disease. Further research is needed to investigate the burden of liver disease in ethnic minority and coastal communities and understand barriers and facilitators to engagement with health care services. More broadly, greater awareness of obesity-related liver disease is needed, which may necessitate a focused public health campaign.

### 3. Liver disease management in primary care

We will build on our HSDR bid to develop education and self-management materials for primary care as it is apparent that education and new clinical pathways for MASLD are urgently needed in primary care. Early discussions with Medicines Innovation (NHS England) revealed their current scoping of early identification methods for ‘at risk’ patients. This highlights the importance and timely nature of MLRA activities identifying cost-effective, scalable methods for prevention and management of MASLD.

## Ethics and consent

No activities within this reported required ethical approval.

## Data Availability

No data were included in this study.
