# Supplementary material for: The Midlands Liver Research Alliance - A partnership to optimise obesity-related liver disease research: targeting areas of high incidence and underserved communities
Source: NIHR Open Res. 2024 Dec 12;4:75. [Version 1] doi: 10.3310/nihropenres.13784.1 (PMC12181761; doi:10.3310/nihropenres.13784.1)
Supplement: Peer review history

# Reviewer report: Reviewer response for version 1

**DOI:** 10.3310/nihropenres.14972.r35250

**Published:** 2025-06-20

**Authors:** Shelley Keating

**Affiliations:** 1 The University of Queensland, St Lucia, Australia

This manuscript by King and colleagues presents the development and early activities of the Midlands Liver Research Alliance (MLRA), a multi-stakeholder partnership aimed at addressing the research and implementation gaps in metabolic dysfunction-associated steatotic liver disease (MASLD). The authors outline the Alliance’s objectives, including the establishment of a PPIE (Patient and Public Involvement and Engagement) stakeholder network, the identification of research priorities, and the mapping of infrastructure and expertise across the region. The manuscript is well written and clearly structured, effectively communicating the processes involved in establishing the partnership, including stakeholder engagement, research strategy development, and capacity mapping.

 The manuscript addresses several critical and timely issues relating to MASLD awareness and management. MASLD represents a growing public health burden, and the initiatives align well with published global priorities for MASLD (both public health and research priorities) and offers a strong rationale for regional collaboration.

 The work provides a valuable foundation for other regional or disease-specific alliances to develop synergies and adopt similar collaborative models.

 Only a few minor comments to help with clarity:

1.  It would be helpful to include a clearer outline of the timelines associated with each major activity. This would aid readers in understanding the pace and sequencing of the partnership’s development.
2. In Activity 1 (paragraph 4), the manuscript mentions training related to PPIE. It would be beneficial to clarify whether this training was intended for academic/industry partners, lived experience partners, or both.
3. The term “multiple long-term conditions” is abbreviated as MLTC in the introduction but not used subsequently. Given the already high number of abbreviations related to MASLD and its care, it may be best to avoid this abbreviation altogether.
4. “AHP” is first abbreviated in paragraph 5 of the background but spelled out in full in point 6 of the objectives. Please ensure consistency by spelling it out at first mention.
5. “GPs” should be spelled out as “general practitioners” at first mention, as this terminology may not be universally recognised. Consider also referencing “primary care practitioners” to enhance global accessibility- as I believe GP’s might not be used consistently worldwide.

Is the work clearly and accurately presented and does it cite the current literature?

Yes

If applicable, is the statistical analysis and its interpretation appropriate?

Not applicable

Are all the source data underlying the results available to ensure full reproducibility?

Yes

Is the study design appropriate and is the work technically sound?

Yes

Are the conclusions drawn adequately supported by the results?

Yes

Are sufficient details of methods and analysis provided to allow replication by others?

Yes

Reviewer Expertise:

Lifestyle care in the management of MASLD and related cardiometabolic diseases.

I confirm that I have read this submission and believe that I have an appropriate level of expertise to confirm that it is of an acceptable scientific standard.

# Reviewer report: Reviewer response for version 1

**DOI:** 10.3310/nihropenres.14972.r35254

**Published:** 2025-05-26

**Authors:** Dimitrios Koutoukidis

**Affiliations:** 1 University of Oxford, Oxford, England, UK

This is a well written and detailed report describing the process and outcomes of a development grant in liver disease. The applicants should be commended to have produced substantial outcomes (3 grant applications to national funding bodies) and have strongly embedded PPI throughout the grant. I have no specific suggestions for improvement.

Is the work clearly and accurately presented and does it cite the current literature?

Yes

If applicable, is the statistical analysis and its interpretation appropriate?

Not applicable

Are all the source data underlying the results available to ensure full reproducibility?

No source data required

Is the study design appropriate and is the work technically sound?

Yes

Are the conclusions drawn adequately supported by the results?

Yes

Are sufficient details of methods and analysis provided to allow replication by others?

Yes

Reviewer Expertise:

Fatty liver disease, obesity, clinical trials

I confirm that I have read this submission and believe that I have an appropriate level of expertise to confirm that it is of an acceptable scientific standard.
